# Hierarchical FBN@Co/CoO heterointerfaces for synergistically tailored microwave attenuation and efficient thermal transport

**DOI:** 10.1016/j.isci.2026.116006

**Published:** 2026-05-20

**Authors:** Jialin Zang, Zhen Lv, Chunpeng Yang, Tianyu Zhang, Yuguang Wang, Dongxu Wang, Haiyan Liu

**Affiliations:** 1 Qiqihar Medical University, Qiqihar, Heilongjiang Province 161006, P.R. China; 2 The Second Affiliated Hospital of Qiqihar Medical University, Qiqihar Medical University, Qiqihar, Heilongjiang Province 161006, P.R. China; 3 College of Pharmacy, Qiqihar Medical University, Qiqihar, Heilongjiang Province 161006, P.R. China

**Keywords:** Physics, Engineering, Materials science

## Abstract

Multifunctional materials integrating electromagnetic wave attenuation and thermal management are crucial for advanced stealth and protection technologies. Herein, rod-like FBN@Co/CoO composites are fabricated via *in situ* growth of uniformly distributed Co/CoO heterointerfaces on fluorinated boron nitride (FBN) surfaces. The optimized FBN@Co/CoO-700 exhibits a minimum reflection loss of −77.40 dB at 4.18 mm and an effective absorption bandwidth of 5.59 GHz, arising from synergistic conduction, polarization, and magnetic losses enabled by Co/CoO heterointerfaces and optimized impedance matching. When incorporated into a UV-curable resin, the composite achieves a thermal conductivity of 0.9964 W m^−1^ K^−1^ at 10 wt. % loading, nearly 4-fold higher than that of the pristine resin, owing to continuous heat-transfer pathways and reduced interfacial thermal resistance. Radar cross section (RCS) simulations further confirm its stealth capability, with RCS values below −10 dB m^2^ within −9°–9° and a minimum of −36.71 dB m^2^. These results highlight the effectiveness of heterointerface engineering for multifunctional electromagnetic-thermal materials.

## Highlights

FBN@Co/CoO heterointerfaces enable absorption (−77.40 dB) and 5.59 GHz bandwidth
• Co/CoO enhances losses and impedance matching
• Incorporation into UV-CR yields 0.9964 W·m^-1^·K^-1^ at 10 wt. % with synergy

## Graphical abstract

**Figure:**
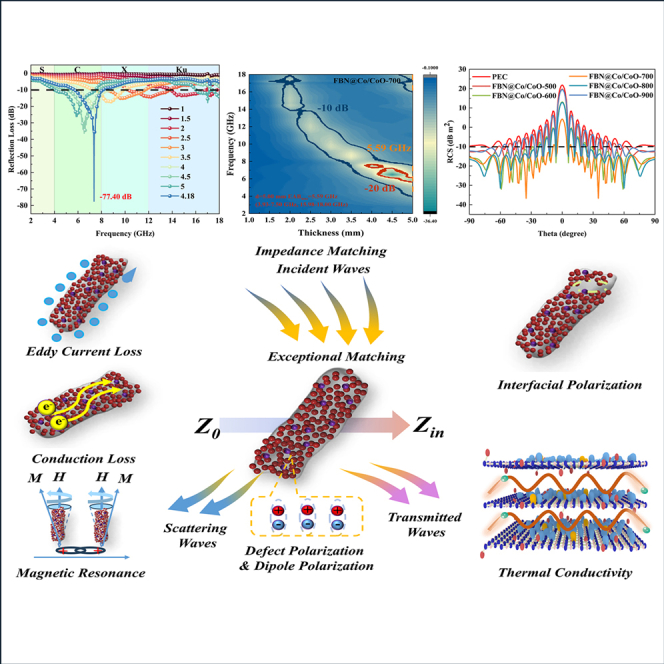

## Introduction

Rapid advances in 5G and emerging 6G communication technologies have greatly improved the efficiency of information transmission and accelerated the widespread integration of intelligent electronic devices into modern infrastructure.^1^^,^^2^^,^^3^ Alongside these developments, the density and operating frequency of wireless systems continue to rise, resulting in pronounced electromagnetic compatibility issues. These challenges are reflected in increased measurement deviations in precision instrumentation and the progressive deterioration of electromagnetic environments in public and industrial settings.^4^^,^^5^ As a result, the development of high-performance microwave absorbing materials (MAMs) has become essential. By coupling dielectric and magnetic loss pathways, such materials can effectively dissipate incident electromagnetic energy. Their core functions are to provide electromagnetic shielding for maintaining the stability of sensitive equipment in fields including healthcare, aerospace, and communications and to regulate electromagnetic fields in order to mitigate the potential biological and environmental impacts associated with high-frequency radiation.^2^^,^^6^ Among the various candidates for microwave absorption (MA) applications, boron nitride (BN) has received considerable attention owing to its layered structure, high thermal conductivity (TC) and outstanding chemical stability.^7^ The combination of BN with magnetic or conductive components such as Fe_3_O_4_ and graphene enables the formation of well-defined heterointerfaces that strengthen interfacial polarization and support synergistic electromagnetic loss processes.^8^ Structural designs that incorporate porous frameworks or core-shell architectures further optimize impedance matching and extend the propagation path of incident electromagnetic waves (EMWs), which collectively enhance attenuation capability.^9^ In addition, the intrinsically high TC of BN contributes to efficient heat dissipation and mitigates thermal accumulation during high-power operation. These attributes make BN a lightweight and durable platform for constructing high-performance MAMs that can meet the stringent requirements of aerospace, 5G, and emerging 6G communication systems.^10^ Such advantages allow BN-based composites to overcome the density constraints and limited environmental stability commonly observed in traditional ferrite and carbon-based systems. Liu et al. fabricated a BN@C composite with a hierarchical pore structure by exploiting the intrinsic porosity of melamine foam and employing a high-temperature-assisted sol-gel method.^11^ Polyvinyl alcohol served as the carbon precursor, while BN acted as the dielectric filler. At optimal thicknesses of 2.5 mm and 1.5 mm, the composite achieved a minimum reflection loss (RL_min_) of −29.12 dB and a maximum effective absorption bandwidth (EAB_max_) of 5.44 GHz. Zhang et al. prepared continuous MXene/BN-zxy composites via a straightforward assembly method, enabling tunable MA properties by adjusting the MXene-to-BN nanosheet ratio.^12^ At a thickness of 4.0 mm, the MXene/BN-101 composite reached an RL_min_ of −20.94 dB, with an EAB_max_ of 9.71 GHz (7.94–17.65 GHz). Despite these advances, BN-based composites still exhibit several critical limitations. Attenuation mechanisms remain relatively restricted, the tunability of absorption characteristics is limited, and processing difficulties often arise during composite fabrication. A major challenge is achieving an appropriate balance between dielectric loss and impedance matching, since overly strong conductive pathways can increase interfacial reflection and reduce effective absorption. The absence of magnetic loss further weakens low-frequency attenuation and typically requires thicker matching layers to achieve the desired performance. Interfacial polarization is also inherently weak because BN primarily acts as a dielectric matrix rather than an active loss component, making it difficult to generate efficient multi-polarization loss. These composites additionally lack pronounced multiple reflection and scattering effects that would extend MA propagation paths and enhance attenuation efficiency. From a structural standpoint, the poor dispersion of BN particles can lead to nonuniform composite architectures, while carbon- and MXene-containing systems are prone to oxidation and therefore suffer degradation in long-term stability. Collectively, these limitations restrict the development of BN-based materials into high-performance MAMs.

Recent developments in metal-metal oxide systems have demonstrated their strong potential in EMW attenuation, primarily through the regulation of dielectric loss, magnetic resonance, and interfacial polarization.^13^^,^^14^ In these hybrid structures, the metallic phase contributes to conduction loss and plasmon resonance, while the oxide phase provides abundant sites for interfacial polarization and facilitates dipole relaxation. The introduction of oxygen vacancies can further enhance dielectric tunability by promoting charge trapping and polarization at defect sites. Carefully designed microstructures, including core-shell configurations and gradient phase distributions, help achieve favorable impedance matching and broaden the EAB. These features collectively make metal-metal oxide systems an effective platform for constructing high-performance MAMs.^15^^,^^16^ For example, Wu et al. optimized the passivation process to induce surface oxidation of Co particles, forming nanoporous Co/CoO structures. After reduction at 200 °C, the Co/CoO composite achieved an RL_min_ of −87.2 dB at a thickness of only 1.0 mm, with an EAB_max_ of 6.2 GHz.^17^ Shen et al. applied a pore-confinement strategy to construct porous Co/CoO/CNF hybrids, where adjusting the acetone content in the spinning solution to 0.30 g yielded a composite with an RL_min_ of −52.2 dB at 3.0 mm and an EAB_max_ of 3.02 GHz.^18^ Li et al. synthesized NiO/Ni nanoparticles with well-defined heterointerfaces on N-doped hollow carbon spheres (NHCS@NiO/Ni) using a SiO_2_ sacrificial template. This composite exhibited an RL_min_ of −44.04 dB at 2.0 mm and an EAB_max_ of 4.38 GHz at 1.7 mm, with performance improvements attributed to the NiO/Ni heterointerfaces, which balanced dielectric loss and optimized impedance matching.^19^ Despite these advances, several key challenges remain. Achieving lightweight structural designs without compromising absorption efficiency is still difficult, and further optimization of microstructural features is required to maximize the contribution of multiple loss mechanisms. The integration of metal-metal oxide heterointerfaces with additional functional phases, particularly lightweight and thermally stable BN-based frameworks, presents a promising pathway for constructing next-generation MAMs with enhanced and tunable performance. Such hybrid architectures have the potential to combine the magnetic and dielectric advantages of metal-metal oxide systems with the structural stability and low density of BN, thereby meeting the stringent requirements of advanced EMW attenuation applications.

Herein, we report the design and fabrication of rod-like fluorinated boron nitride (FBN)@Co/CoO composites via an *in situ* growth strategy, in which uniformly distributed Co/CoO heterointerfaces are anchored onto the FBN surface. This architecture combines the high TC and chemical stability of BN with the multi-mechanism loss characteristics provided by Co/CoO heterointerfaces, enabling simultaneous enhancement of electromagnetic attenuation and thermal management. The optimized FBN@Co/CoO-700 delivers an RL_min_ of −77.40 dB at a thickness of 4.18 mm and an EAB_max_ of 5.59 GHz. Such performance results from the synergistic contributions of conduction loss, interfacial polarization, magnetic loss, and favorable impedance matching. When incorporated into a UV-curable resin (UV-CR) matrix, the composite achieves a TC of 0.9964 W m^−1^ K^−1^ at a loading of 10 wt. % and reaches nearly four times the TC of the pristine matrix, which can be attributed to the formation of continuous heat-transfer pathways and the reduction of interfacial thermal resistance. RCS simulations further demonstrate excellent stealth characteristics with stable performance across a wide range of incident angles. Overall, this work establishes a versatile heterointerface engineering approach for developing lightweight and multifunctional materials that couple efficient EMW attenuation with superior thermal dissipation.

## Results and discussion

### FTIR characterization of FBN@Co/CoO composites

**Figure 1:**
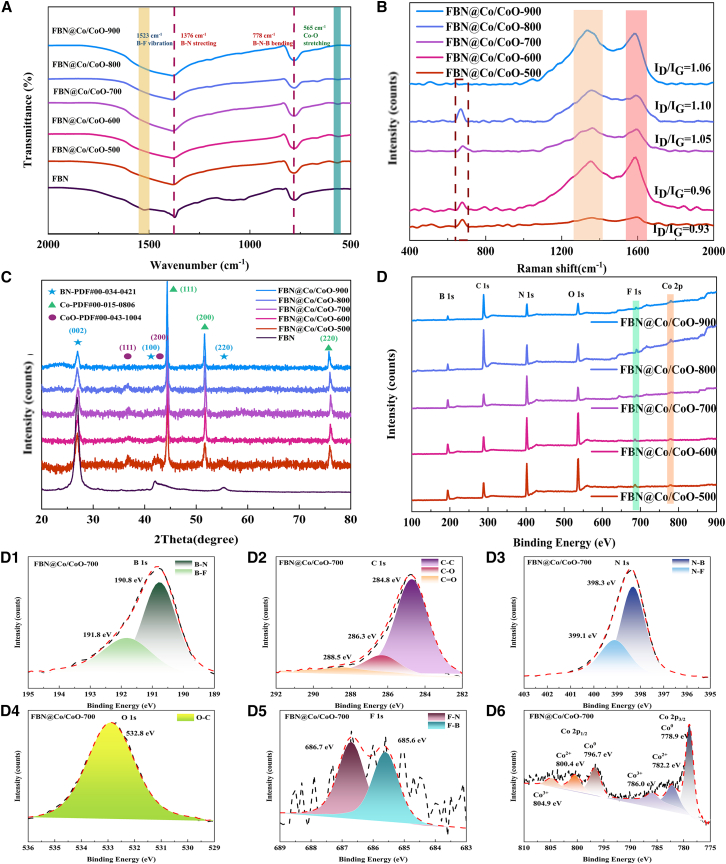
Structural characterization of the FBN@Co/CoO composites (A) FTIR spectra of FBN@Co/CoO composites, (B) Raman spectra of FBN@Co/CoO composites, (C) XRD spectra of FBN@Co/CoO composites, (D) XPS spectra of FBN@Co/CoO composites, and (D1–D6) B 1s, C 1s, N 1s, O 1s, F 1s, Co 2p.

To elucidate the *in situ* formation of Co/CoO species on the FBN surface, Fourier transform infrared (FTIR) spectroscopy was performed to examine the evolution of chemical functionalities. As shown in Figure 1A, all composites exhibit a characteristic band at 1,523 cm^−1^, corresponding to the B-F stretching, along with peaks at 1,376 and 778 cm^−1^ assigned to B-N stretching and out-of-plane B-N-B bending vibrations.^7^ These features confirm that the structural integrity of the FBN framework is well preserved across the investigated temperature range. A band at 565 cm^−1^ assigned to Co-O stretching appears for FBN@Co/CoO-500 to FBN@Co/CoO-800, indicating the formation and gradual stabilization of CoO species on the FBN scaffold.^20^^,^^21^ This band disappears entirely in FBN@Co/CoO-900, suggesting that high-temperature treatment results in complete reduction of CoO to metallic Co. The absence of the Co-O band also implies the loss of Co/CoO heterointerfaces at 900 °C and the collapse of the hybrid CoO phase.

### Raman characterization of FBN@Co/CoO composites

Raman spectroscopy was performed in the range of 400–2,000 cm^−1^ to examine the structural evolution of FBN@Co/CoO composites at different calcination temperatures (Figure 1B). Two characteristic bands appear at approximately 1,360 and 1,590 cm^−1^, corresponding to the D and G bands. The D band is associated with structural disorder, defect sites, and sp^3^-hybridized carbon, whereas the G band arises from the in-plane vibrations of sp^2^-hybridized carbon in graphitic domains. The intensity ratio (I_D_/I_G_) is widely used to evaluate defect density and the abundance of polarization-active sites in carbon-based materials.^18^^,^^22^ The I_D_/I_G_ values show a distinct temperature dependence, increasing from 0.93 at 500 °C to a maximum of 1.10 at 800 °C, followed by a slight decrease to 1.06 at 900 °C. The progressive increase in I_D_/I_G_ from 500°C to 800 °C indicates a higher density of structural defects, which can be attributed to redox reactions associated with the CoO-to-Co transition that locally disrupt the surrounding carbon framework. Additionally, the catalytic activity of metallic Co at elevated temperatures can accelerate carbon decomposition, partial delamination, and lattice restructuring, thereby increasing defect density. The maximum I_D_/I_G_ ratio at 800 °C reflects the greatest degree of disorder, likely resulting from the combined effects of thermal activation, redox-induced structural disruption, and metal-catalyzed carbon degradation. When the calcination temperature reaches 900 °C, the slight decrease in I_D_/I_G_ suggests partial restoration of graphitic order. This behavior may be associated with the complete reduction of CoO to metallic Co, which can promote carbon atom rearrangement and surface graphitization at high temperatures. In addition to the D and G bands, all samples display a Raman peak near 656 cm^−1^, attributed to Co-O vibrations, which confirms the presence of CoO species. The gradual weakening and eventual disappearance of this peak at 900 °C further support the complete reduction of CoO to metallic Co. Interestingly, unlike pure carbon materials where I_G_ typically increases with temperature due to improved atomic ordering, the I_G_ values here exhibit a non-monotonic trend with a decrease at intermediate temperatures. This behavior may arise from the combined effects of fluorine doping and the presence of metal-metal oxide interfaces. Migration of F atoms at elevated temperatures can introduce additional lattice defects and disrupt graphitic ordering. Meanwhile, phase transitions between Co and CoO, together with interfacial interactions with FBN, can introduce local structural strain that suppresses the development of highly ordered graphitic domains. Furthermore, thermal decomposition of carbonaceous species and potential coverage by metal or oxide phases may alter the local Raman scattering environment, making I_G_ quantification more challenging. Complementary X-ray photoelectron spectroscopy (XPS) analyses reveal temperature-dependent changes in chemical states and microstructures, supporting this interpretation. The thermally driven transition from oxidized to metallic states provides important insights into the redox dynamics, catalytic graphitization behavior, and electromagnetic modulation mechanisms within the Co/CoO-carbon hybrid system.

### XRD characterization of FBN@Co/CoO composites

X-ray diffraction (XRD) was performed to analyze the phase composition and crystalline structure evolution of FBN@Co/CoO composites treated at temperatures from 500°C to 900 °C (Figure 1C). All samples show diffraction peaks at 2*θ* = 26.7°, 41.6°, and 55.2°, which are indexed to the (002), (100), and (004) planes of h-BN, respectively (PDF#34-0421).^23^ Additional reflections at 2*θ* = 44.22°, 51.52°, and 75.86° are indexed to the (111), (200), and (220) planes of fcc metallic Co (PDF#00-015-0806),^24^ confirming the presence of crystalline Co. For FBN@Co/CoO-500 to FBN@Co/CoO-800, additional peaks at 2*θ* = 36.50°, 42.40°, and 61.62° correspond to the (111), (200), and (220) planes of CoO (PDF#00-043-1004),^25^ indicating the coexistence of Co and CoO phases. These CoO-related peaks disappear in FBN@Co/CoO-900, suggesting complete reduction of CoO to metallic Co at the highest treatment temperature. These results verify the formation of Co/CoO heterointerfaces and highlight the temperature-dependent phase transformation behavior during thermal treatment.

### XPS characterization of FBN@Co/CoO composites

XPS was used to analyze the surface elemental composition and chemical states of the composites. The survey spectrum (Figure 1D) exhibits B 1s, C 1s, N 1s, O 1s, F 1s, and Co 2p signals, indicating the presence of all expected elements in the composites. In the B 1s spectrum (Figure 1D1), peaks at 190.8 and 191.8 eV are assigned to B-N and B-F bonds, suggesting the presence of fluorinated BN domains. The C 1s spectrum (Figure 1D2) contains components at 284.8, 286.3, and 288.5 eV, corresponding to C-C, C-O, and C=O species, which likely arise from residual carbonaceous layers or surface functional groups. The N 1s spectrum (Figure 1D3) shows peaks at 398.3 and 399.1 eV assigned to N-B and N-F species, indicating the presence of fluorinated nitrogen environments. The O 1s peak at 532.8 eV (Figure 1D4) is attributed to O-C species, which may originate from surface oxidation or residual organics. The F 1s spectrum (Figure 1D5) exhibits peaks at 685.6 and 686.7 eV corresponding to F-B and F-N species, and their relatively low intensity may be related to partial coverage by Co/CoO domains. The Co 2p spectrum (Figure 1D6) shows Co 2p_3/2_ and Co 2p_1/2_ peaks at 778.9 and 796.7 eV characteristic of metallic Co, together with peaks at 782.2 and 800.4 eV attributed to Co^2+^ species and their corresponding satellite features at 786.0 and 804.9 eV. The coexistence of metallic Co and Co^2+^ species indicates the presence of mixed-valence cobalt states, which can promote interfacial polarization and contribute to enhanced electromagnetic response.^24^^,^^25^

### SEM and TEM characterization of FBN@Co/CoO composites

**Figure 2:**
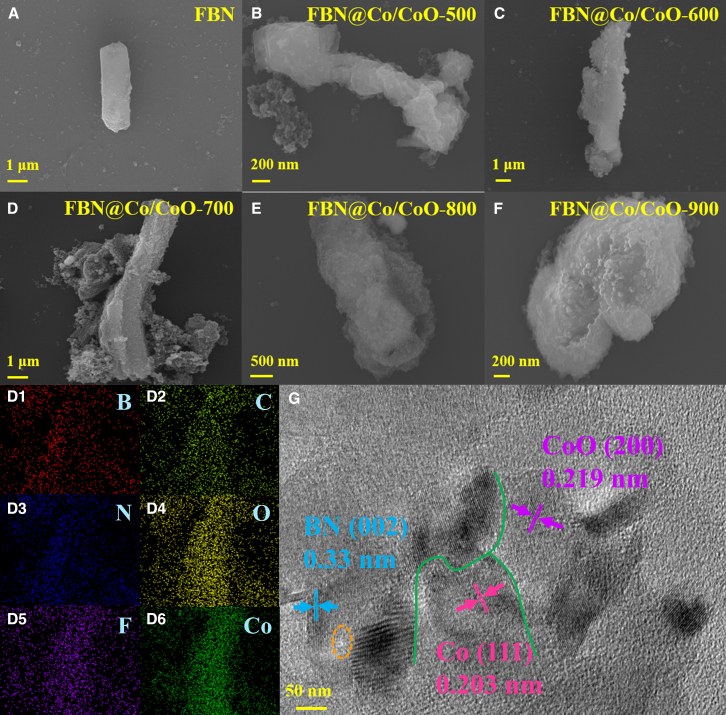
Morphological characterization of the FBN@Co/CoO composites (A–F) SEM images of FBN and FBN@Co/CoO composites, (d1-d6) elemental mapping for FBN@Co/CoO-700, and (g) TEM images of FBN@Co/CoO-700. (Scale bars: 2a: 1 *μ*m; 2b: 200 nm; 2c: 1 *μ*m; 2d: 1 *μ*m; 2e: 500 nm; 2f: 200 nm; 2g: 50 nm).

Figure 2 presents the structural and compositional characteristics of the FBN@Co/CoO composites prepared at different treatment temperatures, as revealed by scanning electron microscopy (SEM), Energy Dispersive Spectroscopy (EDS), and high-resolution transmission electron microscopy (HRTEM) analyses. These characterizations clarify the evolution of microstructure and phase distribution, providing essential evidence for the structure-property correlation discussed below. Figures 2A–2F display the overall morphology of the composites. All samples retain the rod-like architecture inherited from the FBN framework, confirming that the *in situ* growth process does not destroy the structural integrity of the dielectric skeleton. However, notable differences in surface features are observed with increasing treatment temperature. At lower temperatures, Co-containing species appear as relatively sparse and smaller nanoparticles distributed along the FBN surface. As the temperature increases, the density and size of Co/CoO nanoparticles gradually increase, accompanied by partial aggregation and the formation of uneven surface coverage. This non-uniform morphology is likely associated with temperature-dependent nucleation and growth kinetics. Higher thermal energy promotes the reduction of cobalt precursors and accelerates particle coalescence, leading to localized growth and heterogeneous distribution. Meanwhile, surface defects and fluorination-induced polar sites on FBN serve as preferential nucleation centers, further contributing to spatially selective anchoring. High-magnification images reveal that Co/CoO nanoparticles are tightly attached to the FBN surface, indicating strong interfacial interaction. The heterointerface formation becomes more pronounced at optimized temperature, which is expected to enhance interfacial polarization and magnetic-dielectric coupling, thereby influencing electromagnetic attenuation behavior. Elemental distribution was further examined using EDS mapping (Figures 2D1–2D6). B and N elements are homogeneously distributed, consistent with the continuous BN framework. The presence of F confirms successful fluorination, which introduces defect dipoles and polar bonds. Co and O elements are distributed along the FBN rods, indicating the formation of cobalt-containing phases. Although EDS cannot distinguish between metallic Co and CoO phases, it confirms the spatial coexistence of cobalt species on the dielectric matrix. HRTEM analysis (Figure 2G) provides direct evidence of the crystalline structure. A lattice spacing of approximately 0.33 nm corresponds to the BN (002) plane, consistent with layered BN.^26^ A spacing of 0.203 nm is assigned to the Co (111) plane, confirming the presence of metallic Co.^27^ Meanwhile, a lattice spacing of 0.219 nm matches the CoO (200) plane, indicating partial oxidation and the formation of CoO.^28^ The coexistence of Co and CoO lattice fringes demonstrates the formation of Co/CoO heterointerfaces. A slight variation in BN interlayer spacing is observed, which may be attributed to fluorine incorporation and defect formation. Overall, temperature-dependent structural evolution results in tunable nanoparticle size, distribution uniformity, and heterointerface density. These microstructural differences provide the structural basis for the variation in dielectric polarization, magnetic loss, and impedance matching behavior discussed in subsequent sections.

### Magnetic property analysis of FBN@Co/CoO composites

**Figure 3:**
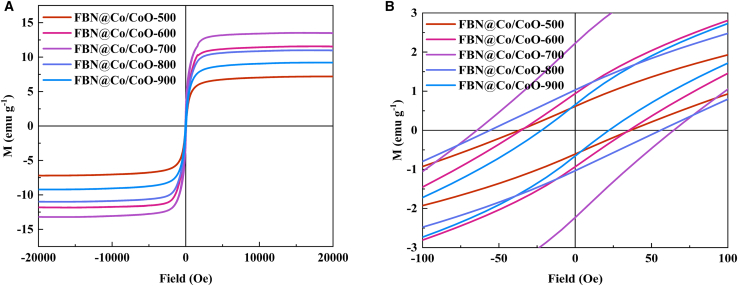
Magnetic properties of the FBN@Co/CoO composites (A) The hysteresis loops of FBN@Co/CoO composites; (B) the coercivity of FBN@Co/CoO composites.

The room-temperature hysteresis loops of FBN@Co/CoO composites annealed at different temperatures (Figure 3) reveal a clear dependence of the ferromagnetic response on thermal treatment. In the high-field region (Figure 3A), FBN@Co/CoO-700 shows the highest saturation magnetization (*M*_*s*_ ≈ 15 emu·g^−1^), exceeding the values of the samples treated at 500, 600, 800°C, and 900 °C. The gradual increase in *M*_*s*_ from 500°C to 700 °C may be related to enhanced reduction of cobalt oxides and the formation of more well-crystallized metallic Co domains, which strengthen ferromagnetic ordering. When the annealing temperature is increased to 800°C and 900 °C, *M*_*s*_ decreases slightly, which may be associated with grain coarsening, partial reoxidation, and a reduction in the interfacial area between Co and CoO, all of which can weaken exchange interactions.

Low-field magnifications (Figure 3B) reveal noticeable differences in coercivity (*H*_*c*_) and loop shape among the samples. The sample annealed at 700 °C exhibits the highest *H*_*c*_ and a more rectangular loop shape, which may reflect enhanced magnetic anisotropy and interfacial interactions between Co and CoO domains. Samples annealed at 500°C and 600 °C show narrower loops and lower *H*_*c*_, which may be associated with smaller Co domains and less-developed crystallinity. Conversely, samples annealed at 800°C and 900 °C exhibit reduced *H*_*c*_, which may be related to grain coarsening and a decrease in interfacial area, potentially weakening domain-wall pinning effects. Slight horizontal shifts observed in some loops may indicate weak exchange-bias-like behavior, although its magnitude cannot be fully resolved from room-temperature hysteresis alone. Collectively, the results show that thermal treatment plays a key role in tuning *M*_*s*_ and *H*_*c*_ by modifying the Co/CoO nanostructure, providing useful guidance for tailoring magnetic behavior in multifunctional electromagnetic materials.

### Electromagnetic performance and related mechanisms of FBN@Co/CoO composites

The RL value was calculated by the following equations^20^^,^^29^:

(Equation 1) $$RL=20\;\log\left|\left(\frac{Z_{in}-Z_{0}}{Z_{in}+Z_{0}}\right)\right|$$

(Equation 2) $$Z_{in}=Z_{0}\left(\sqrt{\frac{\mu_{r}}{\varepsilon_{r}}}\right)\tanh\left[j\left(\frac{2\pi fd}{c}\right)\sqrt{\mu_{r}\varepsilon_{r}}\right]$$

where *c*, *f*, and *d* refer to the light speed, the corresponding frequency, and the matching thickness; *Z*_*in*_ is the normalized input impedance and *Z*_*0*_ means the impedance of free space; and *ε*_*r*_ and *μ*_*r*_ are the complex permittivity and complex permeability, respectively. Normally, when RL < −10 dB, it means that the MAM will absorb more than 90% of the incident EMW.

**Figure 4:**
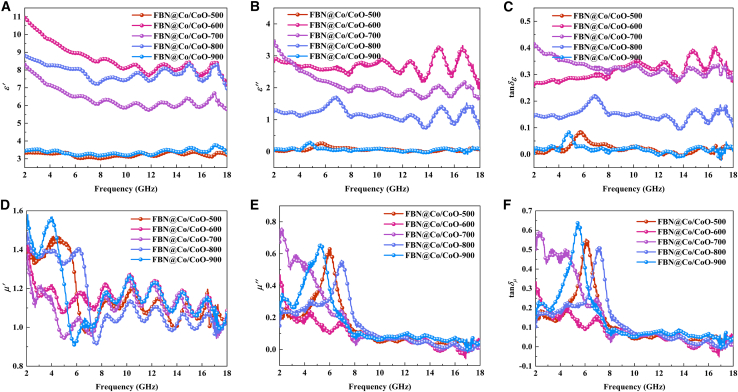
The electromagnetic parameters of FBN@Co/CoO composites (A) *ε′*, (B) *ε′′*, (C) *tanδ*_*ε*_, (D) *μ′*, (E) *μ′′*, and (F) *tanδ*_*μ*_*.*

The dielectric and magnetic responses of FBN@Co/CoO composites in the 2–18 GHz range were investigated through measurements of complex permittivity and permeability.^30^^,^^31^^,^^32^ The real part of permittivity (*ε′*) reflects the ability of a material to undergo polarization and store electrical energy under an alternating electromagnetic field. As shown in Figure 4A, *ε′* of the FBN@Co/CoO composites shows a clear dependence on annealing temperature across the 2–18 GHz range. This behavior is closely related to variations in microstructure and phase composition induced by thermal treatment. The FBN@Co/CoO-600 sample exhibits relatively high *ε′* values, which may originate from abundant Co/CoO interfaces that promote interfacial and dipolar polarization. The noticeable frequency dispersion suggests incomplete formation of continuous conductive pathways and the presence of charge-trapping sites within the composite. As the annealing temperature increases to 700°C and 800 °C, partial reduction of CoO to Co alters the amount of Co/CoO interfacial regions. This reduction in interfacial contrast weakens Maxwell-Wagner-Sillars (MWS) polarization and is reflected in the gradual decrease of *ε′*. At the microscopic mechanism level, MWS polarization arises from charge accumulation at interfaces between phases with different conductivities and permittivities. These regions respond to the applied alternating electric field and contribute to a higher polarization intensity. However, when the CoO phase is partially reduced to Co, the conductivity gradient within the composite diminishes, suppressing charge accumulation at the interfaces and leading to a reduction in polarization strength. An increased proportion of metallic Co may facilitate partial conductive networks within the composite, contributing to higher conduction-related losses. These losses do not increase *ε′* but can accelerate its attenuation at higher frequencies. Additionally, the further formation of the Co phase induces microscopic current loops within the structure, causing the dielectric loss behavior to shift toward conductivity dominance, further suppressing the polarization storage capacity of composite. In the 900 °C sample, the significant reduction of CoO greatly decreases the number of Co/CoO interfaces. As a result, interfacial polarization becomes limited, leading to lower *ε′* values with weaker frequency dispersion. This indicates that the polarization capacity of the composite is significantly constrained. In contrast, although FBN@Co/CoO-500 contains Co/CoO heterointerfaces, its *ε′* is noticeably lower than that of the other composites. This may be due to incomplete development of Co/CoO interfaces at lower temperatures. Lower temperatures may also limit crystallization and interfacial development. The presence of defects may further suppress interfacial polarization strength. Overall, suitable annealing temperatures promote the development of interfacial regions that enhance polarization, whereas excessively high or low temperatures suppress these effects.

As shown in Figure 4B, the imaginary part of permittivity (*ε″*) was examined in the 2–18 GHz range to evaluate the dielectric loss characteristics of the FBN@Co/CoO composites. The evolution of *ε″* is influenced by changes in composition and interfacial features associated with thermal treatment. FBN@Co/CoO-500 exhibits low *ε″* values across the entire frequency range, suggesting limited dielectric loss, likely due to the predominance of insulating CoO and insufficient development of conductive pathways. The weak frequency response also implies limited polarization relaxation processes. In contrast, FBN@Co/CoO-600 shows the highest *ε″* values together with strong frequency dispersion, indicating enhanced dielectric loss. This behavior may arise from more developed Co/CoO interfaces that enhance interfacial relaxation, along with initial formation of conductive regions associated with metallic Co. At 700 °C, *ε″* decreases moderately and shows a more monotonic decline with frequency, indicating a shift toward conduction-related loss mechanisms as metallic Co becomes more prevalent. Although increased metallic Co may enhance conductivity, the reduced number of Co/CoO interfaces likely weakens interfacial polarization. FBN@Co/CoO-800 shows lower *ε″* values with residual fluctuations, suggesting a transitional state in which increased metallic content reduces interfacial polarization, although localized relaxation processes may still occur. Grain coarsening and diminished interfacial contrast may further reduce polarization contributions. FBN@Co/CoO-900 exhibits nearly constant, minimal *ε″* values, indicating that interfacial polarization becomes largely suppressed at high temperatures as CoO is extensively reduced. Overall, the FBN@Co/CoO-600 sample exhibits relatively strong dielectric loss, likely due to a balance among interfacial polarization, dipolar relaxation, and moderate conduction effects.

Annealing in the 600°C–700 °C range provides a favorable balance between dielectric storage and dissipation, as reflected in the dielectric loss tangent (*tan δ*_*ε*_ = *ε″/ε′*), which describes the ratio of energy dissipation to storage capability and serves as a useful parameter for evaluating MA behavior. As shown in Figure 4C, the FBN@Co/CoO-600 exhibits the highest *tan δ*_*ε*_ values, which may result from a combined contribution of interfacial polarization and conduction-related loss. At this temperature, the coexistence of Co and CoO likely provides a considerable number of interfaces that enhance Maxwell-Wagner interfacial polarization. Such interfaces can act as polarization centers that contribute to charge accumulation and relaxation under alternating fields. Meanwhile, the presence of metallic Co may introduce partial conductive pathways that contribute to conduction-related loss. The coexistence of interfacial polarization and moderate conduction yields a balanced dielectric response, contributing to both energy storage and dissipation. Upon annealing at 700 °C, *tan δ*_*ε*_ remains relatively high. At this temperature, further growth of Co may increase overall conductivity. Increased conductivity enhances conduction-related losses, while the relative contribution of polarization becomes less significant. The reduced interfacial contrast may weaken polarization-related contributions. Consequently, *tan δ*_*ε*_ shows a slight decrease as polarization effects are diminished. The composite annealed at 800 °C shows a marked decrease in *tan δ*_*ε*_, suggesting that conduction effects increasingly outweigh polarization contributions. Although conductivity may further increase, excessive metallic character can lead to enhanced reflection and reduced dielectric loss. At both lower (500 °C) and higher (900 °C) annealing temperatures, *tan δ*_*ε*_ is significantly reduced. For FBN@Co/CoO-500, the predominance of CoO limits both conduction and interfacial polarization. In FBN@Co/CoO-900, extensive reduction of CoO leads to high conductivity dominated by free-carrier response. The reduced interfacial contrast and diminished polarization processes contribute to low *tan δ*_*ε*_ values characteristic of highly conductive composites.^33^^,^^34^^,^^35^ In summary, the dielectric loss behavior of the composites is governed by the combined effects of microstructural evolution, phase composition and interfacial characteristics. Annealing at 600°C–700 °C provides a favorable balance between polarization and conduction-related loss mechanisms, contributing to enhanced dielectric attenuation.

Figure 4D illustrates the frequency-dependent evolution of the real part of permeability (*μ′*) for the FBN@Co/CoO composites, reflecting the combined influence of intrinsic magnetic behavior and microstructural changes induced by thermal treatment. The variation in *μ′* is influenced by changes in the Co/CoO phase composition and the development of magnetic interfaces during annealing. For the FBN@Co/CoO-500 and FBN@Co/CoO-600 samples, *μ′* remains relatively stable at low frequencies (2–8 GHz) and gradually decreases at higher frequencies, suggesting limited magnetic loss. This behavior may be related to the predominance of CoO, an antiferromagnetic phase with inherently low permeability. The presence of a small amount of metallic Co may introduce weak magnetic polarization and initial resonance features. At 700°C and 800 °C, increased metallic Co content and the coexistence of Co and CoO create more magnetic interfaces within the composite. These interfaces can contribute to enhanced magnetic anisotropy and may activate multiple resonance processes. As a result, *μ′* pronounced more noticeable fluctuations and enhanced values over the measured frequency range. In the 8–14 GHz region, the oscillatory *μ′* behavior may be associated with multiple magnetic relaxation processes arising from nanoscale Co domains and interfacial interactions between different magnetic phases. In contrast, the FBN@Co/CoO-900 sample shows a noticeable decrease in μ′ at low frequencies (2–6 GHz), followed by irregular oscillations. This behavior may be related to extensive reduction of CoO and the predominance of metallic Co. Although metallic Co contributes to magnetization, the reduced amount of CoO decreases magnetic interfacial contrast, which may weaken interfacial magnetic interactions and associated loss mechanisms. Furthermore, higher temperatures may promote Co aggregation, reducing the density of effective magnetic interfaces and domain-wall-related features, which can further lower permeability.

The imaginary part of the complex permeability (*μ″*) exhibits clear frequency dependence and characteristic fluctuations across samples, suggesting that the magnetic loss behavior is influenced by the differences in phase composition and microstructure. As shown in Figure 4E, all samples display noticeable *μ″* features in the low-frequency region (2–8 GHz), with peak intensity and position varying with annealing temperature. At higher frequencies (8–18 GHz), *μ″* gradually decreases and becomes smoother, consistent with the reduced contribution of magnetic resonance at elevated frequencies. The FBN@Co/CoO-500 sample shows relatively pronounced low-frequency fluctuations, which may be associated with the presence of dispersed Co/CoO phases that create local magnetic inhomogeneities and enable multiple relaxation pathways. At higher frequencies, resonance-related contributions diminish, resulting in smoother *μ″* variations. For the FBN@Co/CoO-600 sample, the lower fluctuation amplitude suggests weaker resonance-related magnetic responses. At higher frequencies, the relatively stable *μ″* profile is consistent with an increased contribution from eddy-current-related loss. The FBN@Co/CoO-700 sample exhibits more pronounced low-frequency *μ″* features, which may arise from increased metallic Co content and stronger magnetic contrast at Co/CoO interfaces, enabling multiple relaxation processes. At higher frequencies, *μ″* becomes smoother as high-frequency resonance contributions weaken. In the FBN@Co/CoO-800 sample, the reduced fluctuation amplitude may be related to grain growth and a decrease in magnetic interface density, leading to fewer active resonance centers. At higher frequencies, *μ″* shows a smoother variation, consistent with the predominance of eddy-current-related loss. The FBN@Co/CoO-900 sample shows minimal low-frequency fluctuations, consistent with the predominance of metallic Co and the reduced contribution of CoO-related magnetic interfaces. The smoother *μ″* curve suggests that magnetic losses at this stage are mainly associated with non-resonant mechanisms, such as eddy-current-related effects, while interfacial-resonance contributions become less significant.

The magnetic loss tangent (*tanδ*_*μ*_ = *μ''/μ′*) of the FBN@Co/CoO composites exhibits clear frequency-dependent behavior (Figure 4F), reflecting the influence of thermal treatment on magnetic dissipation characteristics. Across the measured frequency range, the *tan δ*_*μ*_ curves display alternating fluctuation and smooth regions, suggesting that magnetic loss behavior evolves with changes in the microstructure and phase composition. The FBN@Co/CoO-500 sample exhibits relatively strong *tan δ*_*μ*_ fluctuations, which may arise from magnetic inhomogeneity associated with the coexistence of Co- and CoO-related phases that enable multiple relaxation pathways. In the FBN@Co/CoO-600 sample, the reduced fluctuation amplitude suggests a more uniform magnetic microstructure, accompanied by fewer active magnetic relaxation centers, resulting in a comparatively smoother frequency response. The FBN@Co/CoO-700 sample displays the most pronounced fluctuations, which may be associated with increased metallic Co content and stronger magnetic contrast at Co/CoO interfaces, enabling multiple magnetic relaxation processes. The enhanced fluctuation intensity indicates that several magnetic dissipation pathways may coexist, producing strong frequency-dependent responses. In the FBN@Co/CoO-800 sample, grain growth and reduced interfacial density may contribute to the lower fluctuation amplitude, consistent with fewer active magnetic relaxation centers. The smoother *tan δ*_*μ*_ response suggests that magnetic loss at this stage is mainly contributed by non-resonant mechanisms such as eddy-current-related dissipation. For the FBN@Co/CoO-900 sample, the minimal *tan δ*_*μ*_ fluctuations are consistent with the predominance of metallic Co and the substantial reduction of CoO-related magnetic interfaces. The smooth and continuous *tan δ*_*μ*_ profile indicates that magnetic loss is largely governed by non-resonant mechanisms, such as eddy-current-related dissipation, while interfacial-resonance contributions become less significant. Overall, increasing annealing temperature leads to a progressive transition from heterogeneous Co/CoO composites to Co-dominated structures, accompanied by an evolution of magnetic loss behavior. The *tan δ*_*μ*_ response shifts from strong low-frequency fluctuations to smoother frequency dependence, reflecting the systematic modification of magnetic relaxation pathways with thermal treatment.^36^^,^^37^^,^^38^

**Figure 5:**
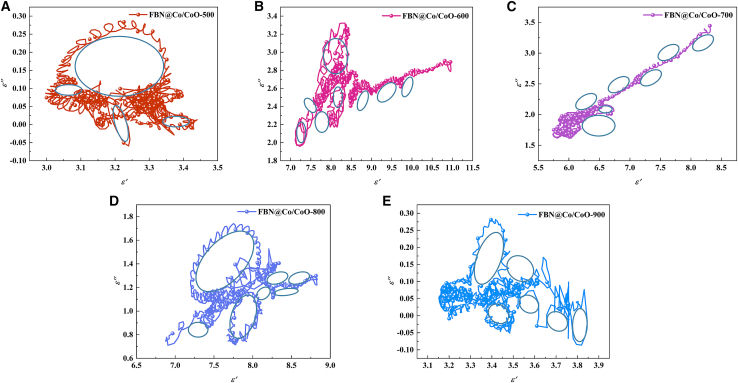
Cole-Cole curves of FBN@Co/CoO composites (A) FBN@Co/CoO-500, (B) FBN@Co/CoO-600, (C) FBN@Co/CoO-700, (D) FBN@Co/CoO-800, and (E) FBN@Co/CoO-900.

Figure 5 presents the corrected Cole-Cole plots of the FBN@Co/CoO composites at different annealing temperatures. All dielectric parameters were re-examined and recalculated to ensure accuracy. The Cole-Cole plots (*ε″* versus *ε′*) are used to analyze dielectric relaxation processes according to Debye relaxation theory, where an ideal single relaxation process generates a semicircular arc. For the FBN@Co/CoO-500 sample, the Cole-Cole plot shows only weak and incomplete semicircular features, accompanied by noticeable distortion. This indicates limited interfacial polarization and relatively low dielectric relaxation intensity at this stage. The absence of well-defined arcs suggests that the heterogeneous interfaces and conductive pathways are not sufficiently developed. At 600 °C and 700 °C, clearer and more distinguishable semicircular arcs emerge, indicating enhanced dielectric relaxation behavior. The increased arc definition reflects the strengthened interfacial polarization associated with the formation of Co/CoO heterointerfaces. These heterogeneous interfaces introduce localized charge accumulation sites, promoting Maxwell-Wagner polarization. Meanwhile, moderate conductivity contributes to improved relaxation intensity without completely dominating the dielectric response. For samples treated at 800 °C and 900 °C, the Cole-Cole plots deviate from ideal semicircular behavior and exhibit more pronounced linear tails in the high-*ε′* region. This feature is characteristic of conductivity-dominated dielectric loss. Excessive growth and partial aggregation of Co-containing phases likely enhance charge transport pathways, leading to increased conduction loss and distortion of classical Debye relaxation arcs. The coexistence of semicircular arcs and linear segments suggests the combined contribution of interfacial polarization, dipolar polarization, and conductivity-related loss. The evolution of these features with annealing temperature reflects the microstructure-dependent dielectric response, which plays a crucial role in regulating impedance matching and microwave attenuation performance.^33^^,^^35^

The enhanced MA performance of the FBN@Co/CoO composites is closely associated with their strengthened dielectric loss, as supported by the preceding permittivity and relaxation analyses. To further clarify the origins of dielectric dissipation, both polarization-related loss and conduction-associated loss were analyzed in detail. According to Debye relaxation theory, the frequency-dependent real and imaginary parts of permittivity (*ε′* and *ε″*) can be expressed as follows^39^^,^^40^:

(Equation 3) $$\varepsilon^{\prime }=\varepsilon_{\infty }+\frac{\varepsilon_{s}+\varepsilon_{\infty }}{1+\tau^{2}\omega^{2}}$$

(Equation 4) $$\varepsilon^{\prime \prime }=\frac{\varepsilon_{s}-\varepsilon_{\infty }}{1+\tau^{2}\omega^{2}}\omega \tau +\frac{\sigma }{{\omega \varepsilon }_{0}}$$

(Equation 5) ω=2πf

where *ε*_*∞*_, *ε*_*s*_, *τ*, *σ*, *ω*, and *f* represent the relative permittivity at infinite frequency, static permittivity, polarization relaxation time, conductivity, angular frequency, and microwave frequency, respectively. By neglecting conduction loss and simplifying the above equation, the following expression can be derived^41^:

(Equation 6) $$\frac{\varepsilon^{\prime \prime }}{f}=2\pi \tau \varepsilon^{\prime }-2\pi \varepsilon_{\infty }$$

**Figure 6:**
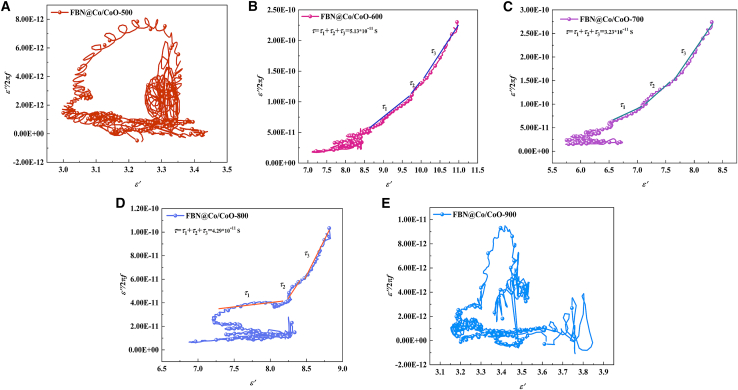
The *ε′′/2πf-ε′* curves of FBN@Co/CoO composites (A) FBN@Co/CoO-500, (B) FBN@Co/CoO-600, (C) FBN@Co/CoO-700, (D) FBN@Co/CoO-800, and (E) FBN@Co/CoO-900.

As expressed in Equation 6, the linear relation between *ε′* and *ε″/2πf* yields the relaxation time *τ*, which characterizes the rate of dielectric polarization response within the material.^42^ The evolution of *τ* with annealing temperature reflects changes in phase composition, interfacial characteristics, and microstructural development within the FBN@Co/CoO system (Figure 6). At 500°C (FBN@Co/CoO-500), the *τ* value is relatively large, which is consistent with the weak dielectric relaxation observed earlier. The limited development of Co/CoO interfaces and the insufficient formation of heterogeneous regions restrict interfacial polarization. As a result, polarization processes proceed more slowly, giving rise to a longer characteristic relaxation time. With increasing annealing temperature, the formation of more well-defined Co/CoO interfaces enhances dielectric heterogeneity and facilitates interfacial polarization, resulting in faster relaxation behavior and a reduced *τ*. Consequently, the total relaxation time (*τ* = *τ*_1_+*τ*_2_+*τ*_3_) gradually decreases, indicating a more rapid and efficient dielectric response. At 900°C, *τ* increases again, which corresponds to the diminished contribution of Co/CoO interfaces due to extensive phase transformation and coarsening of metallic Co domains. These changes weaken interfacial polarization and broaden the relaxation behavior, leading to less efficient dielectric dissipation. The temperature-dependent *τ* evolution underscores the importance of controlling phase composition and interfacial structure to optimize dielectric relaxation and enhance microwave attenuation performance in Co/CoO-based composites.^43^

**Figure 7:**
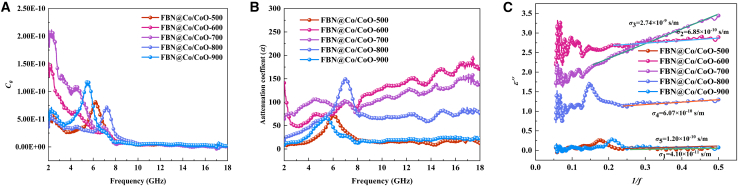
Electromagnetic attenuation and dielectric loss analysis of FBN@Co/CoO composites (A) The *C*_*0*_*-f* curves of FBN@Co/CoO composites, (B) the attenuation coefficient curves of FBN@Co/CoO composites, and (C) the *ε′′-1/f* curves of FBN@Co/CoO composites.

According to Equations 7 and 8, the electromagnetic response of the FBN@Co/CoO composites varies systematically with annealing temperature, as evidenced by the evolution of the eddy current coefficient (*C*_*0*_), attenuation coefficient (*α*), and *ε″-1/f* dispersion behavior.^44^^,^^45^^,^^46^ These variations reflect the combined effects of microstructural evolution, charge transport behavior, and polarization-related loss mechanisms. As shown in Figure 7A, *C*_*0*_ increases significantly at low frequencies for FBN@Co/CoO-600 and FBN@Co/CoO-700. This behavior is consistent with the presence of well-defined Co/CoO interfaces, which enhance dielectric heterogeneity and support interfacial polarization and conductivity-related loss. The decline in *C*_*0*_ at higher frequencies corresponds to the reduced contribution of eddy-current-related loss, which is suppressed by the skin effect at short wavelengths. At higher annealing temperatures (800°C–900°C), the reduction in *C*_*0*_ is associated with the loss of Co/CoO interfaces and the coarsening of metallic Co domains, which weaken interfacial polarization and diminish conductivity-related dissipation. A similar trend is observed for the attenuation coefficient *α* (Figure 7B). The 600°C and 700°C samples exhibit the highest *α* values, reflecting the enhanced contribution of both dielectric and magnetic dissipation under optimized microstructural conditions. The *α* peak appearing near 6–8 GHz is consistent with polarization relaxation processes and frequency-selective magnetic dissipation associated with the Co/CoO heterostructure. At 900°C, *α* decreases sharply due to the disappearance of Co/CoO interfaces and the reduced density of magnetic boundaries, which limit dielectric relaxation and magnetic loss contributions. The *ε*′′-*1/f* dispersion curves (Figure 7C), together with the relation *σ=2πfε′′ε*_*0*_ indicates that the conductivity reaches *σ*_3_ = 2.74 × 10^−9^ S‧m^−1^ for FBN@Co/CoO-700, which is higher than that of the lower-temperature samples. This increase is consistent with the enhanced contribution of the metallic Co phase and the presence of heterogeneous interfaces, both of which facilitate charge transport within the composite. Conversely, the conductivity decreases markedly at 900°C (*σ*_5_ = 1.20 × 10^−10^ S‧m^−1^), which can be attributed to the breakdown of conductive pathways caused by Co domain coarsening and the disappearance of Co/CoO interfaces. These results highlight the importance of controlling phase composition and interfacial structure to regulate conduction loss, interfacial polarization, and magnetic dissipation, thereby optimizing the electromagnetic attenuation performance of FBN@Co/CoO composites.

(Equation 7) $$C_{0}=2\mu^{\prime \prime }{\left(\mu^{\prime }\right)}^{-2}f^{-1}$$

(Equation 8) $$\alpha =\sqrt{2}\frac{\pi f}{c}\times \sqrt{\left(\varepsilon^{\prime \prime }\mu^{\prime \prime }-\varepsilon^{\prime }\mu^{\prime }\right)+\sqrt{{\left(\varepsilon^{\prime \prime }\mu^{\prime \prime }-\varepsilon^{\prime }\mu^{\prime }\right)}^{2}+{\left(\varepsilon^{\prime }\mu^{\prime \prime }\varepsilon^{\prime \prime }\mu^{\prime }\right)}^{2}}}$$

**Figure 8:**
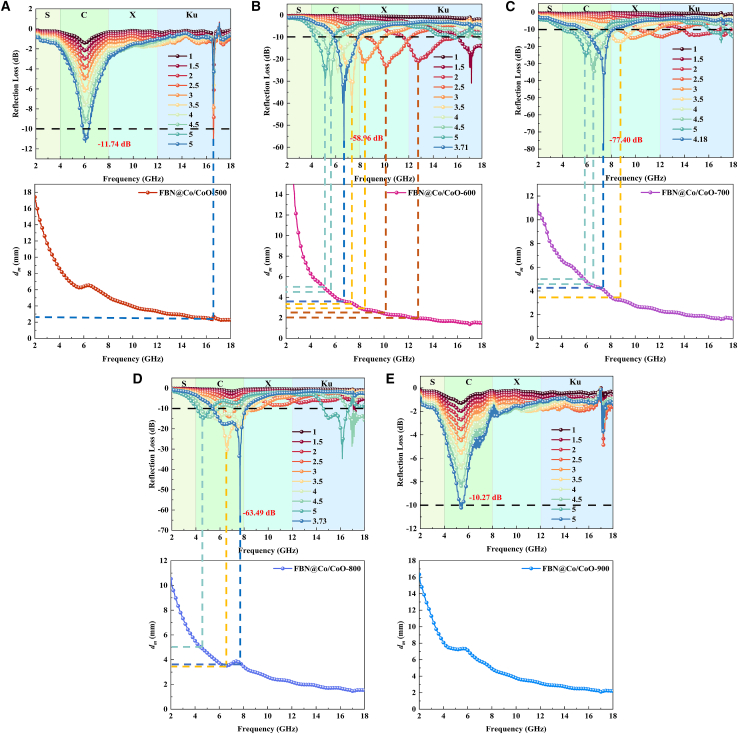
Reflection loss values in the frequency of 2–18 GHz for FBN@Co/CoO composites (A) FBN@Co/CoO-500, (B) FBN@Co/CoO-600, (C) FBN@Co/CoO-700, (D) FBN@Co/CoO-800, and (E) FBN@Co/CoO-900.

Using Equations 1 and 2, the dependence of RL on absorber thickness across the 2–18 GHz range can be determined.^20^^,^^29^ RL_min_ is a key indicator for evaluating MA efficiency. Figure 8 provides a systematic analysis of MA behavior, focusing on two essential aspects. First, it examines how RL varies nonlinearly with absorber thickness. Second, it identifies the frequency-dependent evolution of the optimal impedance-matching thickness. Notably, the RL curves of FBN@Co/CoO-500 and FBN@Co/CoO-900 show minimal dependence on thickness, indicating that impedance mismatch dominates their MA behavior. Figures 8B–8D highlight that FBN@Co/CoO-600, FBN@Co/CoO-700, and FBN@Co/CoO-800 exhibit strong attenuation capability over a wide frequency range. For these samples, RL_min_ values remain below −10 dB across broad spectral regions, covering the C, X, and Ku bands, corresponding to more than 90% energy dissipation. Their broadband adaptability arises from improved impedance matching and multiple loss pathways, consistent with the enhanced relaxation features observed in the Cole-Cole plots. Specifically, FBN@Co/CoO-600, FBN@Co/CoO-700, and FBN@Co/CoO-800 achieve RL_min_ values of −58.96 dB (3.71 mm), −77.40 dB (4.18 mm), and −63.49 dB (3.73 mm), respectively, demonstrating their strong MA capability within the C band. The superior performance of the FBN@Co/CoO composites is associated with the presence of Co/CoO heterointerfaces, which enhance interfacial polarization and contribute to efficient dielectric and magnetic dissipation. The multiscale micro-nano structure further increases the effective dissipation pathways, complementing the interfacial polarization and improving MA performance. The dependence of RL on thickness (*d*_*m*_) and frequency (*f*_*m*_) agrees with the quarter-wavelength (*λ/4*) resonance condition (Equation 9), indicating that appropriate thickness tuning is essential for achieving optimal absorption at target frequencies.^47^

(Equation 9) $$dm=\frac{nc}{4f_{m}\sqrt{\left\|\mu_{r}\right\|\left\|\varepsilon_{r}\right\|}}\left(n=1,3,5,7,\ldots \right)$$

where |*ε*_*r*_| and |*μ*_*r*_| are the measured moduli of *ε*_*r*_ and *μ*_*r*_, respectively.

**Figure 9:**
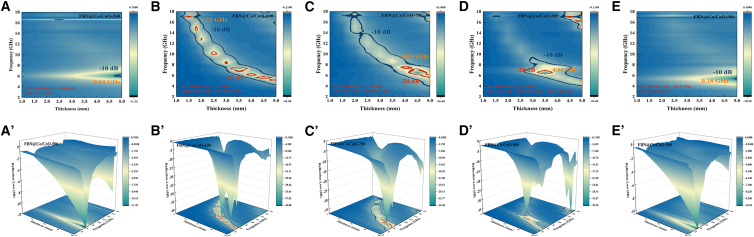
2D and 3D effective absorption bandwidth diagram of 2–18 GHz (A, A′) FBN@Co/CoO-500, (B, B′) FBN@Co/CoO-600, (C, C′) FBN@Co/CoO-700, (D, D′) FBN@Co/CoO-800, and (E, E′) FBN@Co/CoO-900.

To further evaluate the MA performance of the composites, a 3D attenuation-efficiency map was constructed from the thickness-frequency mappings (Figure 9).^48^^,^^49^ As shown in Figure 9A and A*ʹ*, FBN@Co/CoO-500 exhibits high RL values across the entire spectrum, reflecting weak dielectric/magnetic loss capability dominated by the insulating CoO phase. In sharp contrast, the samples annealed at 600°C and 700 °C demonstrate markedly enhanced MA performance over 2–18 GHz, with an EAB exceeding 30% of the measured range (Figures 9B and 9C). FBN@Co/CoO-600 delivers the most balanced absorption behavior, achieving an EAB_max_ of 5.22 GHz at a practical thickness of 1.8 mm. This broadband response (12.52–16.78 GHz and 16.97–17.93 GHz) originates from the well-tuned coexistence of Co and CoO, which strengthens interfacial polarization and magnetic-dielectric coupling (Figures 9B and 9Bʹ). Such phase coexistence enhances magnetic loss and promotes efficient interfacial polarization, thereby improving electromagnetic attenuation. Although FBN@Co/CoO-700 attains a broader EAB of 5.59 GHz, the required thickness of 5 mm compromises its suitability for thin-profile absorber applications (Figures 9C and 9C*ʹ*). At 800 °C, the EAB_max_ decreases to 4.00 GHz at a thickness of 4.93 mm, indicating reduced broadband absorption capability. The absorption splits into three discrete ranges (4.11–5.33 GHz, 14.74–16.76 GHz, and 17.27–18.00 GHz), suggesting that microstructural coarsening and reduced heterointerface density weaken magnetic-dielectric synergy (Figures 9D and 9D*ʹ*). When the annealing temperature reaches 900 °C, CoO is fully reduced to metallic Co, leading to the disappearance of Co/CoO heterointerfaces (Figures 9E and 9Eʹ). This structural transition severely weakens dielectric and magnetic loss pathways, reducing the wideband MA performance to baseline levels, with an EAB_max_ of only 0.18 GHz.

**Figure 10:**
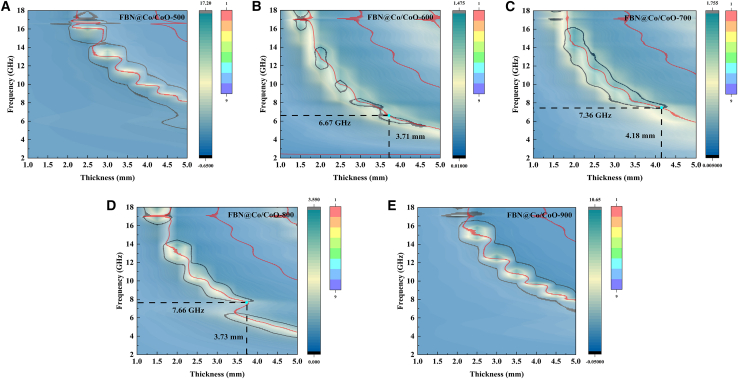
2D schematics of impedance matching of FBN@Co/CoO composites (A) FBN@Co/CoO-500, (B) FBN@Co/CoO-600, (C) FBN@Co/CoO-700, (D) FBN@Co/CoO-800, and (E) FBN@Co/CoO-900.

Figure 10 presents the impedance matching behavior of FBN@Co/CoO composites at different annealing temperatures, revealing how microstructure evolution, dielectric-magnetic loss coupling, and oxidation degree collectively modulate MA performance.^50^ At 500°C (Figure 10A), the composite shows poor impedance matching, indicating that the insufficiently developed Co/CoO heterointerfaces fail to regulate dielectric and magnetic responses, resulting in pronounced wave reflection. Consequently, most incident waves are reflected rather than dissipated. At 600°C (Figure 10B), impedance matching improves markedly, achieving optimal matching at 6.67 GHz with a thickness of 3.71 mm. This improvement arises from the formation of a well-integrated Co/CoO interfacial polarization network, which enhances dipolar and interfacial relaxation processes, enabling deeper wave penetration and greater attenuation. When the annealing temperature increases to 700 °C (Figure 10C), the matching frequency shifts to 7.36 GHz and the optimal thickness increases to 4.18 mm. This shift indicates that moderate oxidation promotes the development of a more continuous conductive network and strengthens Co/CoO interfacial coupling, thereby enhancing dielectric-magnetic synergistic losses. Moreover, the improved balance between permittivity and permeability enhances wave-material coupling and increases attenuation efficiency. At 800°C (Figure 10D), optimal matching shifts to 7.66 GHz with a reduced thickness of 3.73 mm, suggesting that additional oxidation temporarily enhances impedance matching and enables efficient absorption at a thinner profile. This enhancement is associated with increased Co/CoO heterointerface density and strengthened magnetic interactions. At 900 °C (Figure 10E), the impedance matching deteriorates sharply, accompanied by significant wave reflection. This deterioration results from excessive reduction of CoO into metallic Co, which eliminates Co/CoO heterointerfaces and suppresses both conduction loss and interfacial magnetic resonance. Consequently, incident waves cannot effectively enter the material, causing a substantial decline in absorption efficiency. These results underscore that precise control of oxidation degree is essential for tuning dielectric loss, magnetic loss, and impedance matching to realize high-performance MA behavior. As summarized in Table 1, the MA performance of FBN@Co/CoO is benchmarked against representative dielectric absorbers. The comparison demonstrates the superior attenuation capability of the FBN@Co/CoO system, particularly the 700 °C sample, which reaches an RL_min_ of −77.40 dB and an EAB_max_ of 5.59 GHz. This outstanding performance arises from the optimized Co/CoO heterointerfaces, which simultaneously strengthen impedance matching and multi-mechanism wave attenuation. Overall, the FBN@Co/CoO composites exhibit excellent MA efficiency and broad operational bandwidth, highlighting their strong potential for next-generation lightweight MAMs.

**Table 1:** Microwave absorption properties of different Co-based composites

Sample	RL_min_ (dB)	EAB_max_ (GHz)	Reference
Ce/NiCo@C	−67.2	7.12	Yan et al.^1^
CoNi@NC-CoNi@CNTs	−71.7	5.4	Liang et al.^2^
FeCo-Glass	−23.8	5.20	Li et al.^3^
C/Co/Mo2C	−73.46	5.2	Duan et al.^5^
G/C/Fe_3_O_4_/paraffin	−37.2	4.16	Pang et al.^51^
SiC@Co-C	−40.08	4.16	Xiang et al.^52^
Fe_3_O_4_/C	−54.3	5.26	Dou et al.^53^
TiB_2_@BN/PDMS	−31.2	5.12	Liu et al.^54^
NiFe@C	−38.4	4.56	Qu et al.^55^
Ni/MnO/C	−60.1	5.0	Xiang et al.^56^
SiOC(rGO)-SiO2/SiCnws	−31.2	4.08	Feng et al.^57^
Co/C	−47.6	4.9	Zhao et al.^58^
FBN@Co/CoO-700	−77.40	5.59	This work

### RCS simulation performance of FBN@Co/CoO composites

As a key physical parameter in radar stealth technology, the radar scattering cross section (RCS) quantifies the intensity of the echo returned by a target under radar illumination. According to Equation 10, RCS is defined as the projected area of an equivalent isotropic scatterer that generates the same echo response.^59^

(Equation 10) $$\sigma_{RCS}\left(dB\;m^{2}\right)=10\;\log\;{\left(\frac{4\pi S}{\lambda^{2}}\left|\frac{E_{s}}{E_{i}}\right|\right)}^{2}$$

where *S*, *λ*, *E*_*s*_, and *E*_*i*_ represent the area of the simulation model, wavelength of electromagnetic wave, and electric field intensities of the scattered and incident waves, respectively.

**Figure 11:**
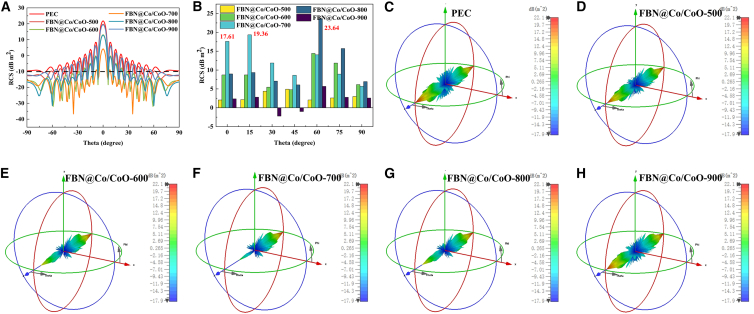
Radar scattering response and RCS reduction analysis of FBN@Co/CoO composites (A) The simulated RCS values at scattering angles from −90° to 90°, (B) RCS reduction values of Perfect Electric Conductor (PEC) and PEC coated with FBN@Co/CoO composites, and (C–H) 3D radar scattering signals of PEC and FBN@Co/CoO composites.

Using CST Studio Suite 2021 and RCS modeling, the electromagnetic wave-scattering modulation capabilities of FBN@Co/CoO composites annealed at different temperatures were systematically evaluated. The simulations reveal temperature-dependent dielectric-magnetic coupling behavior and corresponding MA mechanisms.^60^ As shown in Figure 11A, the angular RCS distributions (−90°–90°) demonstrate that FBN@Co/CoO-700 exhibits the most pronounced stealth performance among all samples. Its RCS value remains below −10 dB m^2^ within −9° < *θ* < 9°, and reaches a minimum of −36.71 dB m^2^, indicating excellent suppression of backscattered electromagnetic waves. In comparison, FBN@Co/CoO-600 shows moderate attenuation capability, whereas FBN@Co/CoO-900 displays markedly weaker performance due to the loss of Co/CoO heterointerfaces. As shown in Figure 11B, FBN@Co/CoO-700 exhibits a 19.36 dB m^2^ reduction at *θ* = 15°, confirming its strong angular stealth capability. Interestingly, FBN@Co/CoO-800 achieves an even larger attenuation of 23.64 dB m^2^ at *θ* = 60°, attributed to its anisotropic scattering behavior. The 3D RCS patterns (Figures 11C–11H) reveal a clear evolution in the scattering characteristics of the composites with increasing annealing temperature. The phase distributions transition from symmetric to asymmetric forms, particularly for FBN@Co/CoO-600 and FBN@Co/CoO-700. This transition arises from improved impedance matching and the emergence of multi-scale scattering pathways during pyrolysis. Correlating structure with performance reveals that polarization relaxation at Co/CoO heterointerfaces, combined with the multiscale porous architecture generated during high-temperature treatment, plays a dominant role in modulating scattering behavior. These factors synergistically enhance electromagnetic energy dissipation through coupled physicochemical mechanisms. By establishing a quantitative correlation between heterostructure regulation and electromagnetic response, the study underscores the critical importance of heterointerface engineering for high-efficiency MAMs. The experimental results confirm that precise control of pyrolysis temperature enables a tunable balance between dielectric and magnetic losses, thereby overcoming the inherent limitations of single-component absorbers. These insights provide a theoretical basis for designing high-performance MAMs and offer guidance for developing next-generation intelligent absorbers with programmable electromagnetic responses.

### Thermal conductivity of FBN@Co/CoO composites

The FBN@Co/CoO composites efficiently convert incident microwave energy into heat; however, the associated localized thermal accumulation may pose risks to device stability.^61^ To alleviate this issue, thermally conductive components were incorporated into the system to enhance internal heat transport and dissipation, thereby mitigating thermal hotspots and improving thermal reliability.^62^ Moreover, the incorporation of UV-CR provides additional advantages. Its intrinsically low thermal expansion coefficient helps maintain structural integrity at elevated temperatures, whereas its rapid curing kinetics and favorable rheological properties facilitate the uniform dispersion of thermally conductive fillers within the matrix. This optimized microstructure promotes the formation of continuous thermal pathways, thereby enhancing the overall thermal management performance of the composite.^63^

**Figure 12:**
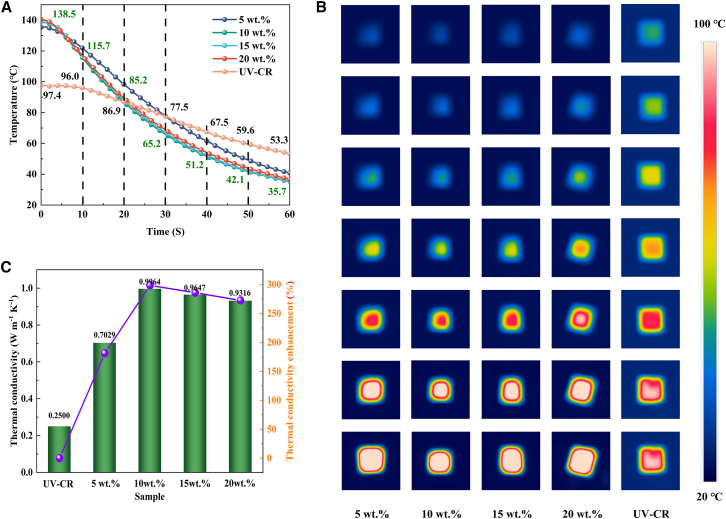
Thermal conductivity of the composites (A) Temperature-time curves of FBN@Co/CoO-700/UV-CR composites, (B) infrared thermal images of FBN@Co/CoO-700/UV-CR composites, and (C) thermal conductivity and thermal conductivity enhancement of FBN@Co/CoO-700/UV-CR composites with different filler contents. (Data are represented as mean ± SEM.)

To assess the thermal transport capability of FBN@Co/CoO-700/UV-CR composites with different filler loadings, the samples were heated on a 100 °C hot plate for 60 s, and their transient heat dissipation behavior was recorded via infrared thermography. As shown in Figure 12A, pure UV-CR exhibited a relatively low surface temperature of 97.4°C after heating, notably lower than that of the composites containing FBN@Co/CoO fillers. It also showed a slower cooling rate, retaining a surface temperature of 53.3°C after 60 s. In contrast, all composite samples (5–20 wt. %) exhibited markedly enhanced heat dissipation, with surface temperatures rising to approximately 138 °C post heating. This improvement indicates that incorporating FBN@Co/CoO-700 into the UV-CR matrix promotes the formation of continuous thermal-conduction networks, thereby enhancing heat uptake and transport efficiency within the composite. The composite with 5 wt. % filler exhibited slightly reduced thermal dissipation relative to higher-loading samples. Increasing the filler loading beyond 10 wt. % produced no further noticeable enhancement in thermal transport. This saturation behavior arises because reduced inter-filler spacing at higher loadings initially promotes network formation, but once a percolated pathway is established, additional filler contributes marginally. Once a percolated thermal network forms at approximately 10 wt. %, additional fillers provide minimal benefit, as the existing pathways already support efficient phonon transport. Furthermore, although fillers are uniformly dispersed, their random orientation hinders the development of anisotropic conductive pathways, particularly perpendicular to the heat-flow direction, where thermal bottlenecks are more likely to persist. Interfacial thermal resistance remains another critical limiting factor. Even without agglomeration, the increased number of filler-matrix interfaces at higher loadings introduces additional boundary resistance, thereby impeding overall heat transfer. Overall, incorporating FBN@Co/CoO-700 substantially enhances the TC of the UV-CR matrix by facilitating internal heat transport and accelerating thermal diffusion to the surroundings. The improved interfacial compatibility between Co/CoO heterointerfaces and the UV-CR matrix strengthens phonon coupling across filler-matrix boundaries, thereby promoting effective thermal bridging. This synergistic interfacial effect mitigates localized heat accumulation and promotes spatially uniform thermal diffusion, collectively contributing to the composite’s superior thermal-regulation capability. The infrared thermal images in Figure 12B visually corroborate the enhanced heat-dissipation capability of the composites. Consistent with these observations, the measured TC values (Figure 12C) increase from 0.2500 W m^−1^ K^−1^ for pristine UV-CR to a peak of 0.9964 W m^−1^ K^−1^ at 10 wt. %, corresponding to an enhancement exceeding 298.56%. This substantial enhancement highlights the critical role of FBN@Co/CoO-700 in improving thermal-transport efficiency, which arises from both the intrinsically high TC of the fillers and their synergistic integration within the composite network.

**Figure 13:**
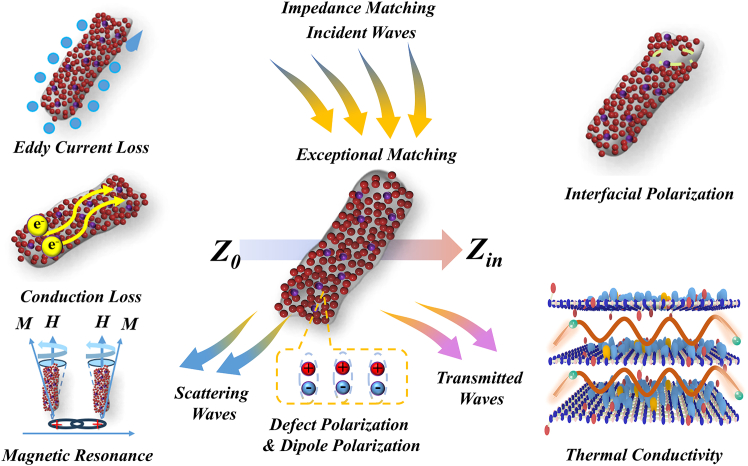
Microwave absorption and thermal conductivity mechanism for FBN@Co/CoO composites

Figure 13 systematically elucidates the impedance-matching characteristics and multichannel energy dissipation mechanisms of FBN@Co/CoO composites. The well-balanced impedance matching enables efficient coupling of incident microwaves into the composite, suppressing front-surface reflection and providing the prerequisite for effective internal attenuation. Once the waves enter the interior, energy dissipation proceeds through several synergistic dielectric and magnetic pathways. The intrinsic structural defects of FBN, together with F-doping-induced polarization centers, activate defect and dipole polarization, thereby contributing to frequency-dependent dielectric relaxation. The Co/CoO heterointerfaces create abundant interfacial polarization sites, where dielectric discontinuity induces localized electric-field distortion, promoting charge accumulation, interfacial relaxation, and enhanced polarization loss. Additionally, conduction loss originates from the metallic Co domains, where continuous carrier hopping and scattering dissipate electromagnetic energy as Joule heat. Magnetic loss is dominated by the magnetic anisotropy and exchange coupling at Co/CoO interfaces, where the ferromagnetic Co phase supports natural and exchange resonance, while the antiferromagnetic CoO domains contribute to hysteresis loss and interfacial pinning. Furthermore, eddy-current loss arises from circulating currents induced within the conductive network, which consume electromagnetic energy through Ohmic dissipation. Ultimately, the cooperative action of these dissipation pathways regulates the competition between scattering, absorption, and transmission, maximizing internal energy attenuation and enabling superior MA performance. Thermal conduction in FBN@Co/CoO composites arises from the synergistic coupling of high-TC fillers and interfacial thermal bridging. In this system, FBN contributes intrinsically high in-plane TC, while Co/CoO nanoparticles anchored along its surfaces function as interconnective thermal bridges between adjacent FBN units. This hierarchical configuration minimizes inter-filler spacing and establishes continuous heat-conduction pathways throughout the composite. At the microscopic scale, heat is mainly transferred through phonons propagating along the crystalline FBN framework. Metallic Co domains further facilitate heat transfer through electron-phonon coupling, while the CoO phase acts as a stabilizing interfacial layer that suppresses thermal boundary resistance. The intimate contact between Co/CoO and FBN reduces phonon scattering at the matrix-filler interface, enabling more efficient energy transfer. Consequently, the composite forms a dual-scale conduction network composed of (i) a surface-mediated thermal-bridging pathway constructed by Co/CoO interconnections and (ii) a long-range phonon-transport pathway embedded within the FBN skeleton. The interplay of these pathways accounts for the significant enhancement in TC, even at relatively low filler loadings. By elucidating the synergistic interplay between electromagnetic dissipation pathways and interfacial thermal bridging, this study provides a mechanistic framework for the rational design of high-performance MAMs with integrated thermal-management capability.

In summary, rod-like FBN@Co/CoO composites featuring uniformly distributed Co/CoO heterointerfaces anchored on the FBN surface were successfully constructed via an *in situ* growth strategy. Benefiting from the rational regulation of phase composition and interfacial architecture, the optimized FBN@Co/CoO-700 exhibits outstanding MA performance, achieving an ultralow RL_min_ of −77.40 dB at a thin matching thickness of 4.18 mm and a broad EAB_max_ of 5.59 GHz. Such exceptional performance originates from the synergistic integration of conduction loss, polarization loss, and magnetic loss induced by Co/CoO heterointerfaces, together with significantly improved impedance matching. Beyond electromagnetic attenuation, FBN@Co/CoO-700 demonstrates remarkable thermal transport capability when incorporated into a UV-CR matrix. At a low filler loading of 10 wt. %, the TC of the composite increases dramatically from 0.2500 to 0.9964 W m^−1^ K^−1^, corresponding to nearly a 4-fold enhancement. This improvement is primarily attributed to the formation of interconnected thermal-conduction pathways and enhanced interfacial compatibility between the Co/CoO heterointerfaces and the polymer matrix, which collectively facilitate phonon transport and suppress interfacial thermal resistance. RCS simulations further confirm the excellent stealth characteristics of FBN@Co/CoO-700, with RCS values maintained below −10 dB m^2^ within a narrow angular range (−9° < *θ* < 9°) and reaching a minimum of −36.71 dB m^2^. Even at an oblique incidence angle of 15°, a substantial RCS reduction of 19.36 dB m^2^ is preserved, highlighting robust angular stability. Collectively, these results demonstrate that heterointerface engineering provides an effective strategy for integrating efficient microwave attenuation, enhanced thermal dissipation, and stable stealth performance within a single material system, offering a promising pathway toward next-generation electromagnetic protection and stealth technologies.

### Limitations of the study

Despite the promising electromagnetic attenuation and thermal management performance of the FBN@Co/CoO composites, several limitations remain. The MA and TC evaluations were primarily conducted under laboratory conditions, and their stability and reliability in complex service environments (e.g., high humidity, temperature fluctuation, or corrosive media) have not yet been fully verified. The interfacial loss mechanisms were interpreted mainly from macroscopic electromagnetic parameters, while direct experimental evidence at the micro/nanoscale—particularly regarding dynamic interfacial coupling and magnetic-dielectric synergy—remains limited. In addition, the composite performance was assessed at relatively low filler loadings and in a single polymer matrix, and broader applicability across different matrices and processing conditions requires further validation. Finally, although RCS simulations indicate favorable stealth potential, experimental far-field measurements and large-scale structural demonstrations are still needed to confirm practical performance.

## Resource availability

### Lead contact

Requests for further information and for resources, materials, and data should be directed to and will be fulfilled by the lead contact, Dr. Zhen Lv (Email: pangmax27@163.com).

### Materials availability

This study did not generate new unique materials, cell lines, or biological resources. All synthesized samples are available from the lead contact upon reasonable request.

### Data and code availability

The data that support the findings of this study are available from the corresponding author upon reasonable request.

Code: This paper does not report original code. Other Items: Any additional information required to reanalyze the data reported in this paper is available from the lead contact upon request.

## Acknowledgments

This material is based upon work supported by the “double first-class” characteristic discipline project of oral biomedical materials research and development and personalized manufacturing in Heilongjiang Province. Thanks to the Heilongjiang Provincial Key Laboratory of Pharmacy for their support. Thanks to the Key Lab of Oral Biomedical Materials and Clinical Application of Heilongjiang Province for their support.

This work was financially supported by the Basic Scientific Research Operating Expenses Program for Provincial Undergraduate Universities of the Heilongjiang Provincial Department of Education (2023-KYYWF-0885, 2022-KYYWF-0819) and the Joint Guidance Project of the Qiqihar Science and Technology Plan (LSFGG-2022038). We also appreciate the support from the Leading Talent Team in Pharmacy of Qiqihar.

## Author contributions

J.Z.: writing – original draft, conceptualization, investigation, supervision, funding acquisition. Z.L.: Writing – review and editing, conceptualization, supervision. C.Y.: investigation. T.Z.: investigation. Y.W.: investigation. D.W.: investigation. H.L.: investigation. All authors have read and approved the final manuscript.

## Declaration of interests

The authors declare that they have no known competing financial interests or personal relationships that could have appeared to influence the work reported in this paper.

## STAR★Methods

### Key resources table

**Table:** 

REAGENT or RESOURCE	SOURCE	IDENTIFIER
**Chemicals, peptides, and recombinant proteins**		

Boric acid	Anhui Zesheng Technology Co., Ltd. (Shanghai, China)	CAS: 10043-35-3
Melamine	Anhui Zesheng Technology Co., Ltd. (Shanghai, China)	CAS: 108-78-1
N,N'-dimethylformamide (DMF)	Anhui Zesheng Technology Co., Ltd. (Shanghai, China)	CAS: 68-12-2
Ammonium fluoride	Anhui Zesheng Technology Co., Ltd. (Shanghai, China)	CAS: 12125-01-8
1,3,5-benzenetricarboxylic acid	Anhui Zesheng Technology Co., Ltd. (Shanghai, China)	CAS: 554-95-0
Cobaltous nitrate hexahydrate	Anhui Zesheng Technology Co., Ltd. (Shanghai, China)	CAS: 10026-22-9
Ethylene glycol	Anhui Zesheng Technology Co., Ltd. (Shanghai, China)	CAS: 107-21-1

**Software and algorithms**		

Origin 2021	Origin 2021	https://www.originlab.com
MDI Jade 9	Materials Data	https://www.materialsdata.com/prodjd.html
OMNIC	Thermo Fisher Scientific	https://www.thermofisher.cn/cn/zh
Avantage	Thermo Fisher Scientific	https://www.thermofisher.cn/cn/zh
CST Studio Suite 2021	Computer Simulation Technology AG	https://www.3ds.com

### Method details

#### Preparation of FBN@Co/CoO composites

**Scheme 1:**
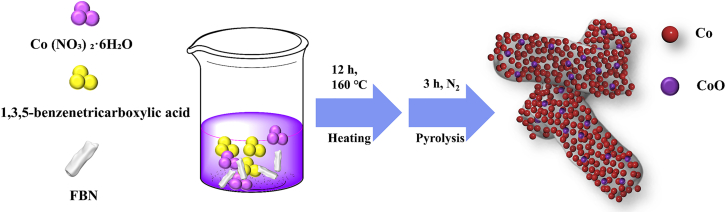
The preparation process of the FBN@Co/CoO composites

The synthesis of h-BNNR and FBN followed a well-established protocol.^7^ Specifically, 1 mmol Co (NO_3_)_2_·6H_2_O and 0.5 mmol 1,3,5-benzenetricarboxylic acid were dissolved in a 70 mL mixed solvent of DMF, ethanol, and deionized water (volume ratio: 12:1:1) under vigorous stirring for 30 min, yielding a dark red solution. Subsequently, 0.2 g of FBN was introduced, and the mixture was magnetically stirred at 600 rpm for an additional 10 min to ensure thorough dispersion. The resulting solution was then transferred into a 100 mL Teflon-lined autoclave and subjected to hydrothermal treatment at 160°C for 12 h. After naturally cooling to room temperature, the product was collected via centrifugation, thoroughly washed with ethanol and deionized water, and dried at 60°C for 12 h, yielding the FBN@Co precursor. This precursor was then annealed under an N_2_ atmosphere at 500 °C, 600 °C, 700 °C, 800 °C, and 900 °C for 3 h with a controlled heating rate of 5 °C·min^-1^, leading to the formation of FBN@Co/CoO composites, which were designated as FBN@Co/CoO-500, FBN@Co/CoO-600, FBN@Co/CoO-700, FBN@Co/CoO-800, and FBN@Co/CoO-900, respectively. The preparation process of the FBN@Co/CoO composites is illustrated in Scheme 1.

#### Preparation of FBN@Co/CoO-700/UV-CR composites

To prepare the composite samples, FBN@Co/CoO-700 was incorporated into the UV-CR matrix at loading levels of 0, 5, 10, 15, and 20 wt.%. Each mixture was subjected to 30 minutes of ultrasonication to ensure uniform filler dispersion, then cast onto glass substrates and cured under UV irradiation for 30 min per cycle, repeated four times to ensure complete polymerization. The resulting films were mechanically polished with 1500-grit sandpaper and cut into cubic specimens with a footprint of 100 mm^2^ for subsequent thermal characterization.

#### Characterizations

X-ray photoelectron spectroscopy (XPS) was performed on a Thermo Scientific ESCALAB 250Xi spectrometer (Thermo Fisher Scientific, USA) equipped with a monochromatic Al K*α* source (*hν* = 1486.6 eV). Measurements were conducted under ultrahigh vacuum (UHV) conditions (base pressure < 1 × 10^-9^ mbar). Survey spectra were acquired at a pass energy of 100 eV, and high-resolution spectra at 20 eV. The energy scale was calibrated using the C 1s peak at 284.8 eV, with an overall system energy resolution better than 0.5 eV, verified from the Ag 3d_5/2_ linewidth. Spectral deconvolution was performed in CASA XPS (v2.3.19) using a Shirley background and Gaussian-Lorentzian (GL 70:30) line shapes. Spin-orbit doublets and satellite peaks were fitted simultaneously under consistent constraints of peak separation and intensity ratio, ensuring physical accuracy. Variations in linewidths among components were rationalized by distinct chemical environments. Quantitative atomic ratios were corrected for photoionization cross sections and analyzer transmission efficiency. Measurements were carried out on powdered samples mounted on conductive carbon tape. X-ray diffraction (XRD) was performed using a Bruker D8 Advance diffractometer (Bruker AXS, Germany) equipped with a Cu K*α* radiation source (*λ* = 1.5406 Å, photon energy = 8.04 keV) operating at 40 kV and 40 mA. Measurements were conducted in Bragg-Brentano geometry with a 1D LynxEye detector, over a 2*θ* range of 10-80° with 0.02° step size and 10° min^-1^ scan rate. The instrumental angular resolution was approximately 0.01°. Phase identification and lattice-parameter refinement were carried out using MDI JADE 9 software, with crystallite size estimated via the Scherrer equation. Data were analyzed with an uncertainty of ±0.02° in peak position. Raman spectroscopy was performed on a Thermo Scientific DXRxi Raman imaging microscope (Thermo Fisher Scientific, USA) using a 532 nm laser (2 mW output power) for excitation, operated over 400-2000 cm^-1^ with a spectral resolution of ∼1 cm^-1^. A 50× objective (NA = 0.75) and CCD detector were used for collection. Calibration was performed using a silicon standard at 520 cm^-1^, and spectra were processed in OMNIC v9.2 software using baseline correction, cosmic ray removal, and Lorentzian peak fitting. Measurements were conducted at room temperature under ambient air. Transmission electron microscopy (TEM) and high-resolution TEM (HRTEM) were performed on a JEOL JEM-2100F microscope (JEOL, Japan) operated at an accelerating voltage of 200 kV. Bright-field (BF) and high-resolution imaging were used to examine morphology and lattice structure, respectively. Selected area electron diffraction (SAED) patterns were obtained for phase identification. Samples were prepared by ultrasonically dispersing powders in ethanol and drop-casting onto carbon-coated copper grids. Images were analyzed using Digital Micrograph (v3.43) for lattice spacing and fast Fourier transform (FFT) filtering. Surface morphology and elemental distribution were examined using a TESCAN MIRA LMS field-emission scanning electron microscope (TESCAN, Czech Republic) operated at 5-15 kV under high vacuum, primarily in secondary-electron (SE) mode. Elemental composition was characterized using an Oxford SmartEDX system (Oxford Instruments, UK) equipped with AZtec v5.0 software. Elemental mapping and point analyses were performed, and standardless quantitative results were calibrated with a pure copper standard. The quantitative uncertainty was within ±2 at%. Magnetic properties of the FBN@Co/CoO composites were measured using a Lake Shore 7404 vibrating-sample magnetometer (VSM) (USA) at room temperature, with an applied field range of ±20 kOe. The thermal conductivity (TC) of the composites was evaluated using a DRL-III thermal conductivity tester (China) combined with a Fotric 246M infrared thermal imaging camera (China). The FBN@Ni-NiO-700/UV-CR composites were fabricated using an integrated washing and UV-curing system (Creality UW-02, China). The relative complex permittivity and permeability of the composite-wax composites were determined using a vector network analyzer (Agilent E5071C, USA) in the frequency range of 2-18 GHz. The as-prepared composites were fabricated by uniformly mixed with paraffin matric (mass fraction of 50 wt.%) and further pressed into a coaxial ring with an outer diameter of 7 mm and an inner diameter of 3.04 mm.

### Quantification and statistical analysis

There are no quantification or statistical analyses to include in this study.
